# A LuxR Homolog in a Cottonwood Tree Endophyte That Activates Gene Expression in Response to a Plant Signal or Specific Peptides

**DOI:** 10.1128/mBio.01101-16

**Published:** 2016-08-02

**Authors:** Amy L. Schaefer, Yasuhiro Oda, Bruna Goncalves Coutinho, Dale A. Pelletier, Justin Weiburg, Vittorio Venturi, E. Peter Greenberg, Caroline S. Harwood

**Affiliations:** a University of Washington, Seattle, Washington, USA; b Oak Ridge National Laboratory, Oak Ridge, Tennessee, USA; c International Centre for Genetic Engineering and Biotechnology, Trieste, Italy

## Abstract

Homologs of the LuxR acyl-homoserine lactone (AHL) quorum-sensing signal receptor are prevalent in *Proteobacteria* isolated from roots of the Eastern cottonwood tree, *Populus deltoides*. Many of these isolates possess an orphan LuxR homolog, closely related to OryR from the rice pathogen *Xanthomonas oryzae*. OryR does not respond to AHL signals but, instead, responds to an unknown plant compound. We discovered an OryR homolog, PipR, in the cottonwood endophyte *Pseudomonas* sp. strain GM79. The genes adjacent to *pipR* encode a predicted ATP-binding cassette (ABC) peptide transporter and peptidases. We purified the putative peptidases, PipA and AapA, and confirmed their predicted activities. A transcriptional *pipA-gfp* reporter was responsive to PipR in the presence of plant leaf macerates, but it was not influenced by AHLs, similar to findings with OryR. We found that PipR also responded to protein hydrolysates to activate *pipA-gfp* expression. Among many peptides tested, the tripeptide Ser-His-Ser showed inducer activity but at relatively high concentrations. An ABC peptide transporter mutant failed to respond to leaf macerates, peptone, or Ser-His-Ser, while peptidase mutants expressed higher-than-wild-type levels of *pipA-gfp* in response to any of these signals. Our studies are consistent with a model where active transport of a peptidelike signal is required for the signal to interact with PipR, which then activates peptidase gene expression. The identification of a peptide ligand for PipR sets the stage to identify plant-derived signals for the OryR family of orphan LuxR proteins.

## IMPORTANCE

We describe the transcription factor PipR from a *Pseudomonas* strain isolated as a cottonwood tree endophyte. PipR is a member of the LuxR family of transcriptional factors. LuxR family members are generally thought of as quorum-sensing signal receptors, but PipR is one of an emerging subfamily of LuxR family members that respond to compounds produced by plants. We found that PipR responds to a peptidelike compound, and we present a model for Pip system signal transduction. A better understanding of plant-responsive LuxR homologs and the compounds to which they respond is of general importance, as they occur in dozens of bacterial species that are associated with economically important plants and, as we report here, they also occur in members of certain root endophyte communities.

## INTRODUCTION

The fast-growing Eastern cottonwood tree, *Populus deltoides*, possesses a distinct microbiota of endophytic (dominated by *Gamma*- and *Alphaproteobacteria*) and rhizosphere-associated (dominated by *Acidobacteria* and *Alphaproteobacteria*) bacteria (1). We have shown that acyl-homoserine lactone (AHL)-type quorum-sensing (QS) genes are prevalent in the genomes of *Proteobacteria* isolated from *Populus* roots (2). Quorum sensing is a cell-to-cell signaling system that allows bacteria to control the expression of genes in a cell density-dependent manner. The AHL QS regulatory circuits include both signal synthases (encoded by *luxI-*type genes) and signal receptors (encoded by *luxR-*type genes) (3, 4). Often the AHL synthase and its coevolved receptor genes are linked on the chromosome, but some *luxR* homologs are not linked to a *luxI* gene. Such *luxR* genes are termed orphans or solos (2, 5) and are abundant in genomes of bacteria isolated from *P. deltoides* (2). Some of the better-studied orphan LuxRs respond to AHLs made by another paired LuxI-LuxR system present in the same cell (6) or by AHLs exogenously provided from neighboring bacteria (7, 8), while the recently described orphan LuxRs from *Photorhabdus* species have been shown to detect endogenous, non-AHL metabolites (9, 10).

Interestingly, many of the *Populus* root isolates encode members of a particular subfamily of LuxR orphan receptors (2) that are responsive to plant-derived chemical elicitors rather than AHLs (reviewed in references 5, 11, and 12). Apparently these LuxR homologs sense their plant host, rather than a QS signal (12, 13). Compared with the AHL-responsive LuxRs, little is known about how these plant-responsive homologs function, and the plant-associated compounds that serve as their ligands have yet to be identified. The best-studied examples are from plant-pathogenic members of the genus *Xanthomonas* (14–17), but similar systems are found in other plant-associated bacteria (11–13), including plant symbionts (18) and biocontrol agents (12). LuxR homologs from several of these bacteria have been shown to activate the transcription of adjacent genes annotated as encoding proline iminopeptidases (*pip* genes). The *pip* genes have been implicated as virulence factors in some bacteria (14, 15). To distinguish the plant-responsive LuxR homologs from the AHL-responsive LuxR homologs, we refer to this subfamily of regulators as OryR regulators, because *X. oryzae* OryR was one of the earliest described plant-responsive LuxR homologs (16).

Here, we describe an OryR regulator that we name PipR, encoded in the *Populus* root endophyte *Pseudomonas* sp. strain GM79 (2), a member of the *Pseudomonas fluorescens* subfamily (19, 20). The genes flanking *pipR* are predicted to encode peptidases and an ATP-binding cassette (ABC) peptide transporter. We show that, similar to *X. oryzae* OryR, PipR activates the transcription of a flanking peptidase gene in response to plant leaf macerates but not in response to AHLs. PipR also responded to protein hydrolysates and a specific peptide (Ser-His-Ser) to activate the expression of the flanking peptidase gene. We show that the PipR response requires the ABC transporter and is modulated by the adjacent peptidase enzymes, perhaps forming a feedback loop. We propose that because we have identified a specific signal molecule, the *Pseudomonas* sp. GM79 PipR system can serve as a model for molecular analyses of the plant-responsive OryR family of signaling systems, which are found in a large number of diverse, plant-associated bacteria.

## RESULTS

### GM79 possesses an *oryR* homolog, which is flanked by peptidase genes.

The genome of *Pseudomonas* sp. GM79 (21) contains two orphan *luxR* homologs (2), *PMI36_01833* and *PMI36_04623.* The polypeptide encoded by *PMI36_01833* is a homolog of the PpoR orphan from *Pseudomonas putida*, which responds to the AHL signal, 3-oxo-hexanoyl-l-homoserine lactone (3-oxo-C6-HSL) (2, 22). The other *luxR* homolog, *PMI36_04623*, is predicted to be a member of the OryR subfamily of plant-responsive LuxR homologs, based on its amino acid sequence and the context of neighboring *pip* genes (2, 12). Like other OryR-type polypeptides, PMI36_04623 has a tryptophan in place of a tyrosine that is conserved in the AHL-responsive LuxR homologs, but unlike the *Xanthomonas* and *Ensifer* OryR homologs, a conserved tryptophan residue remains unchanged (see Fig. S1 in the supplemental material) (reviewed in reference 12).

**FIG 1:**
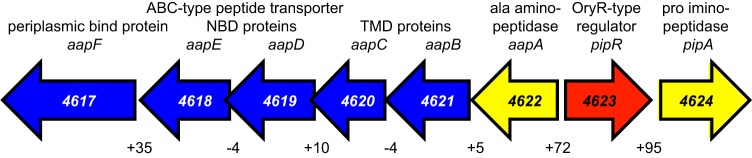
*Pseudomonas* sp. GM79 genomic region surrounding the *oryR* homolog *pipR* (red, *PMI36_04623*). The region includes genes predicted to encode peptidases (yellow, *PMI36_04622* and *PMI36_04624*) and an ABC-type peptide transporter (blue, *PMI36_04617-04621*). There are five peptide transporter genes coding for one periplasmic binding protein, two nucleotide-binding domain (NBD) proteins, and two transmembrane domain (TMD) proteins. The positive numbers below the genes indicate the number of bases in the intergenic region separating the genes; a negative number indicates there is overlap of the two genes.

All known *oryR* homologs are flanked by at least one gene annotated as a proline iminopeptidase gene (*pip*) (15). In GM79, the *oryR* homolog is flanked by two genes predicted to encode proline iminopeptidases (http://img.jgi.doe.gov) (21) (Fig. 1), in a genomic arrangement similar to that of the *oryR* homolog (*nesR*) in *Ensifer meliloti* (18). To confirm whether the genes flanking the GM79 *oryR* homolog actually code for peptidases, both enzymes were purified as hexahistidine-tagged fusion proteins and assayed for their ability to cleave N-terminal amino acid residues from a variety of fluorescent (β-naphthylamide) and chromogenic (*p*-nitroanilide) substrates (Table 1). The PMI36_04622 enzyme was most active in cleaving an N-terminal alanine, while the PMI36_04624 enzyme exhibited good activity in cleaving N-terminal proline and, to a slightly smaller degree, alanine. Both enzymes had moderate activity with hydroxy-proline-, serine-, and methionine-linked substrates, while little-to-no peptidase activity was observed with histidine-, glutamic acid-, and lysine-linked substrates (Table 1). Based on the substrate specificities exhibited by the purified GM79 enzymes, we propose naming *PMI36_04622* and *PMI36_04624 aapA* for alanine aminopeptidase and *pipA* for proline iminopeptidase, respectively.

**TABLE 1:** Substrate specificities of purified His_6_-PipA and His_6_-AapA enzymes

Substrate	Mean activity ± SD^a^	
	His_6_-PipA	His_6_-AapA
l-Proline-β-naphthylamide	100.0 ± 13.4	9.7 ± 0.8
l-Alanine-β-naphthylamide	79.4 ± 12.5	331.1 ± 39.9
l-Hydroxy-proline-β-naphthylamide	30.4 ± 1.3	23.5 ± 2.6
l-Serine-β-naphthylamide	21.5 ± 2.6	12.0 ± 0.5
l-Leucine-β-naphthylamide	7.7 ± 1.8	2.1 ± 1.0
l-Histidine-β-naphthylamide	ND	2.9 ± 0.8
l-Glutamic acid-β-naphthylamide	ND	ND
l-Proline-*p*-nitroanilide	0.72 ± 0.01	0.006 ± 0.001
l-Methionine-*p*-nitroanilide	0.17 ± 0.02	0.160 ± 0.021
l-Lysine-*p*-nitroanilide	ND	ND

a Enzyme (PipA [PMI36_04624] and AapA [PMI36_04622]) purification and assay conditions are described in Materials and Methods; the results are the mean activities from 4 to 8 assays. Naphthylamide substrate results were measured as relative fluorescence units (RFU) per min per mg of protein and normalized to the activity exhibited by His_6_-PipA with L-proline-β-naphthylamide as the substrate. Nitroanilide substrate results are reported as millimoles cleaved per min per mg of protein. ND, not detected (not above the background of the no-added-enzyme control).

### A bioassay for the plant-derived signal.

**FIG 2:**
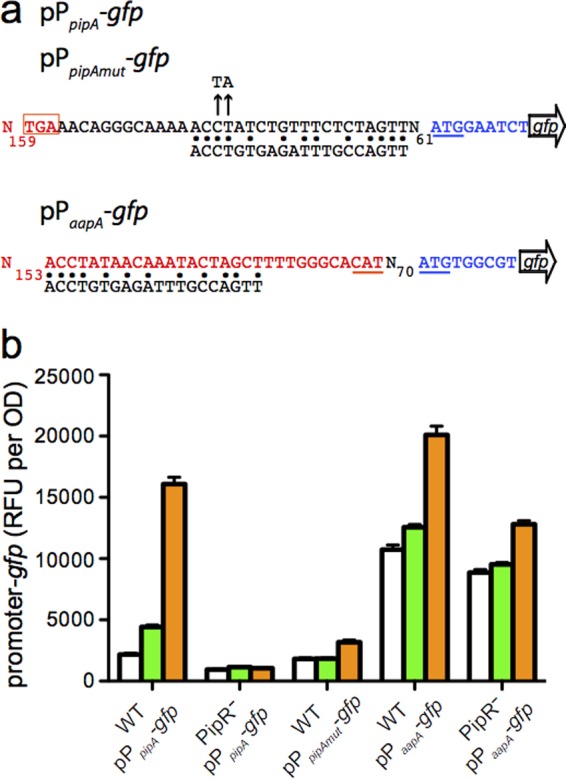
Activities of *pipA* and *aapA* promoters in cells grown in the presence of leaf macerates or peptone. (a) DNA sequences of the *pipA* and *aapA* promoter regions cloned into HindIII-BamHI sites of the promoter-*gfp* transcriptional fusion plasmid pPROBE-NT (see Materials and Methods; see also Table S1 in the supplemental material). Blue letters indicate the first three codons of the *pipA* (top) or *aapA* (bottom) ORF, black letters indicate the intergenic, noncoding sequences, and red letters show the *pipR* DNA sequence (top, 3′ end of *pipR*; bottom, noncoding strand of the 5′ end of *pipR*). The 20-bp DNA sequence below both promoter sequences is the *Xanthomonas oryzae* OryR-binding sequence (24); bases identical to those in the *pipA* or *aapA* (overlapping the *pipR* ORF) promoter regions are indicated by black dots. Translation start codons (or their complements) are underlined, and the *pipR* stop codon is boxed. The two mutations in the predicted PipR-binding site of pP*_pipAmut_-gfp* (Materials and Methods) are indicated by the black arrows (top, CT changed to TA). (b) Activity of the indicated promoter-*gfp* probe in GM79 wild type (WT) or the *pipR* mutant (PipR^−^) grown in the presence of water control (white bars), 0.25% leaf macerates (green bars), or 0.5% peptone (orange bars). The data are the mean relative fluorescence units (RFU) per optical density (OD) unit from six replicates, and the error bars represent the standard deviations.

To aid in the identification of the predicted plant-derived signal for *Pseudomonas* sp. GM79, we required a promoter that uses the PMI36_04623 OryR homolog for activation. In other systems, the *pip* gene adjacent to the *oryR-*type gene is often under OryR control (14–16). In the presence of the plant-derived ligands, the OryR homologs are believed to bind inverted repeat DNA elements (23) and activate gene transcription. The gene encoding the *Pseudomonas* sp. GM79 OryR homolog is also upstream from a proline iminopeptidase gene (*pipA*), and thus, we have named it *pipR* (Fig. 1). Previously, we reported that an inverted repeat sequence centered −71.5 bp upstream from the translational start site of the GM79 *pipA* gene matched the published DNA-binding site for *X. oryzae* OryR in 13 of 20 bases (2). We created the reporter plasmid pP*_pipA_-gfp* (see Materials and Methods; see also Table S1 in the supplemental material), which contains a transcriptional fusion of the GM79 *pipA* promoter with the green fluorescent protein gene (*gfp*) (Fig. 2a). We hypothesized that the GM79 *pipA* promoter would be active when GM79 (pP*_pipA_-gfp*) was grown in the presence of plant macerates but not when grown with AHLs (16). For these experiments, we grew the GM79 (pP*_pipA_-gfp*) strain in minimal medium (see Materials and Methods) to avoid the potential activation of the PipR system, as has been reported for OryR when *X. oryzae* is grown in rich medium even in the absence of rice macerates (24). We tested six AHL signals (see Materials and Methods) with various side-chain lengths and substitutions and found that, even at relatively high concentrations (1 µM), pP*_pipA_-gfp* expression was not higher than in the controls with only water added. Our initial experiments using *Populus* leaf macerates were unsuccessful, as the growth of our reporter strain was inhibited. *Populus* leaves are known to contain high concentrations of phenolics (25), which can be toxic to bacteria. Therefore, we utilized a protocol to remove the growth inhibition activity from the *Populus* leaf macerates (see Materials and Methods). The partially purified leaf macerates, referred to hereinafter as leaf macerates, induced pP*_pipA_-gfp* activity by a modest but reproducible twofold (Fig. 2b). These results are quantitatively similar to those observed with *X. oryzae* (24)*.*

### PipR can respond to protein hydrolysates and specific tripeptides.

**FIG 3:**
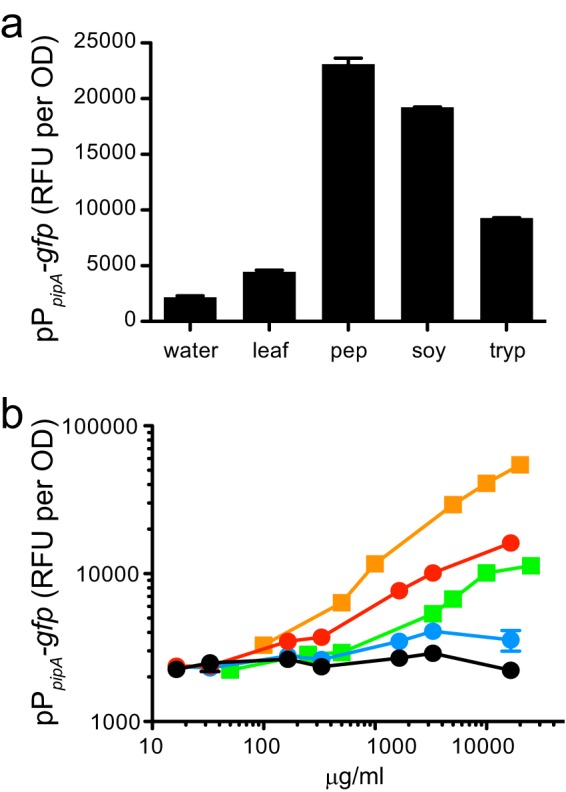
The pP*_pipA_-gfp* reporter is activated by the addition of *Populus* leaf macerates, protein hydrolysates, and the SHS tripeptide. (a) Activity of the pP*_pipA_-gfp* reporter in wild-type cells grown in the presence of the following: water control, 0.5% leaf macerates (leaf), 1% Bacto-peptone (pep), 1% Bacto-soytone (soy), and 1% Bacto-tryptone (tryp). (b) Dose-response for pP*_pipA_-gfp* activation by peptone (orange squares), leaf macerates (green squares), or SHS (red circles), HSS (blue circles), or SSH (black circles) tripeptide. The leaf macerate and peptone concentrations indicated were calculated by using the original concentrations prior to the cleanup protocol (Materials and Methods). The data are the mean RFU per OD unit from six replicates, and the error bars represent the standard deviations.

Because the genes flanking *pipR* are involved in peptide metabolism, we hypothesized that the plant signal may be peptidelike. We tested a variety of peptide-rich protein hydrolysates and found several that could activate the expression of the pP*_pipA_-gfp* gene fusion (Fig. 3a). Enzymatic digests of animal tissue (Bacto-peptone), soybean meal (Bacto-soytone), and pancreatic digest of casein (Bacto-tryptone) each activated pP*_pipA_-gfp* expression.

Because protein hydrolysates are rich in small peptides (26), we screened a small library of compounds (268 dipeptides and 14 tripeptides) that are available as part of the Biolog phenotype microarrays for microbial cells for the ability to activate pP*_pipA_-gfp*. Five dipeptides induced GFP above background levels: Gly-Cys, His-Gly, His-Pro, His-Ser, and Ser-Pro. Small amounts (1 mg) of His-Ser, His-Pro, and Ser-Pro are available for purchase (AnaSpec), so we retested these dipeptides using known concentrations, but only His-Ser had appreciable pP*_pipA_-gfp* reporter activity (data not shown). We purchased a larger amount (100 mg) of His-Ser from another vendor (Sigma-Aldrich) but were surprised to find that this material failed to activate our reporter. Mass spectrometry analysis confirmed that the primary species (100% relative abundance) found in both samples was His-Ser (M + H = 243.1090, 0 ppm); however, a minor species (~5% relative abundance) with a mass consistent with a tripeptide compound containing one histidine and two serine residues (M + H = 330.1407, 0 ppm) was found only in the active sample (AnaSpec). To test the hypothesis that this minor tripeptide species was responsible for the pP*_pipA_-gfp* reporter activation, we tested all three possible tripeptide variations (SSH, SHS, and HSS) (Fig. 3b). Two of the tripeptides, SSH and HSS, had little to no activity (Fig. 3b, black and blue circles) even at the highest concentration tested (16.5 mg/ml or 50 mM). However, the SHS tripeptide showed a moderate level of pP*_pipA_-gfp* reporter expression (Fig. 3b, red circles), but only at relatively high concentrations (≥0.33 mg/ml or 1 mM). We suspect that the signal(s) present in the leaf macerate is not the SHS tripeptide, as LuxR homologs usually respond to nM (or lower) levels of their ligand (27): at 1 mM concentrations, SHS would be easily detected by mass spectrometry of plant macerates, and we cannot find it there. However, the pP*_pipA_-gfp* reporter expression with the specific SHS tripeptide is further evidence that the native ligand may be peptidelike.

### The PipR protein is the receptor for the response to plant macerates and the transcription activator of *pipA* expression.

**FIG 4:**
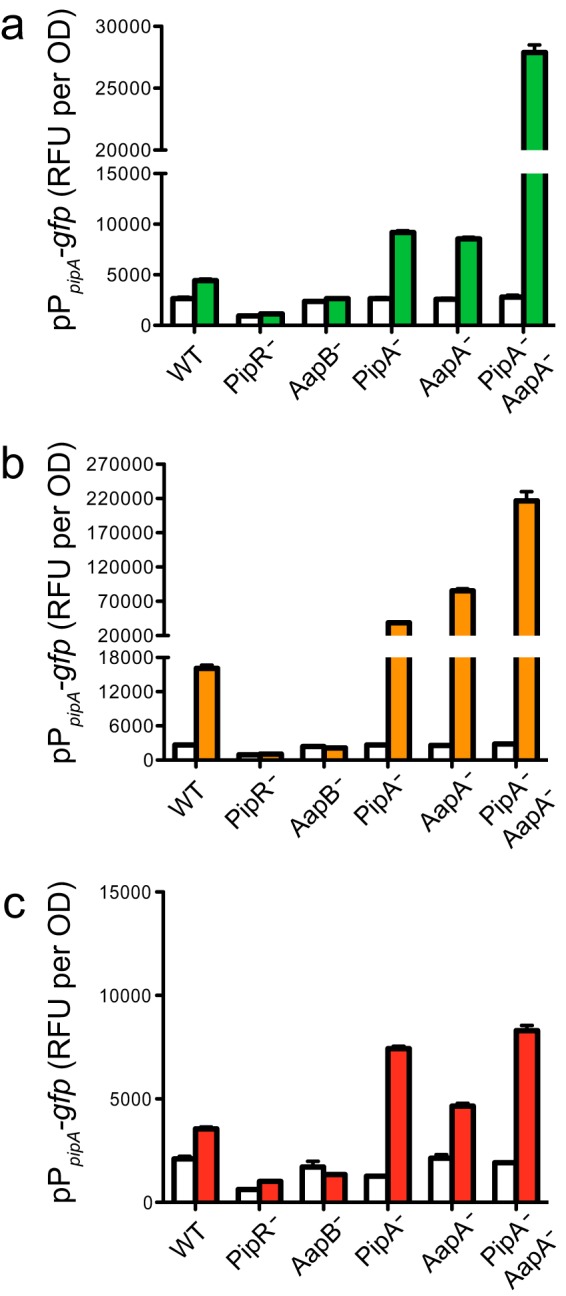
Influence of mutations in the *pipR*-flanking genes on pP*_pipA_-gfp* activity. In all panels, the strains are wild type (WT), *pipR* (*PMI36_04623*) mutant (PipR^−^), *aapB* (*PMI36_04621*) TMD transporter mutant (AapB^−^), *pipA* (*PMI36_04624*) mutant (PipA^−^), *aapA* (*PMI36_04622*) mutant (AapA^−^), and *pipA aapA* (*PMI36_04624* and *PMI36_04622*) double mutant (PipA^−^AapA^−^). Data are the activities of the pP*_pipA_-gfp* reporter grown in the water control (white bars) or in the presence of the following additions: 0.25% leaf macerates (green bars) (a), 0.5% peptone (orange bars) (b), and 1 mM (0.03%) SHS tripeptide (red bars) (c). The data are the RFU per OD unit from six replicates, and the error bars represent the standard deviations.

Leaf macerate, peptone, and the SHS tripeptide all failed to activate the expression of the pP*_pipA_-gfp* reporter in a *pipR* deletion mutant, thus implicating the PipR protein as the signal receptor (Fig. 4). To confirm whether the DNA region of dyad symmetry predicted to bind the PipR protein was required for pP*_pipA_-gfp* activation, we mutated two conserved bases known to be important for binding of LuxR homologs (28) to create pP*_pipAmut_-gfp* (see Table S1 and Fig. S2a in the supplemental material) and found that PipR protein-dependent transcription from the *pipA* promoter was abolished (Fig. 2b). The *pipR* mutation was complemented by expressing *pipR* from a plasmid—although overexpression of *pipR* on a multicopy plasmid resulted in high GFP expression levels even in the absence of signal (see Fig. S2a).

There is also a potential PipR-binding site centered −91.5 bases upstream from the ATG start of the *aapA* gene (2), although this sequence overlaps the 5′ coding region of the *pipR* gene (Fig. 2a). To test whether the *aapA* gene was also under control of PipR, we created an *aapA* promoter reporter plasmid, pP*_aapA_-gfp* (see Table S1 and Fig. S2a in the supplemental material). The basal *gfp* expression levels of pP*_aapA_-gfp* were about five times higher than those of pP*_pipA_-gfp* in wild-type cells. The addition of leaf macerates had a very small effect, but peptone stimulated pP*_aapA_-gfp* expression by about 1.5-fold (Fig. 2b). The expression of pP*_aapA_-gfp* in a PipR deletion strain was reduced in cells grown in the presence of peptone (Fig. 2b). These results indicate that PipR strongly controls downstream *pipA* expression and has a small but measurable effect on *aapA* expression.

### A mutation in the putative ABC transporter gene *aapB* abolishes induction of pP*_pipA_-gfp* by plant macerates, peptone, and SHS tripeptide.

The *aapA* gene and the downstream ABC-type transporter genes, now named *aapB*, -*C*, -*D*, -*E*, *and -F*, are likely cotranscribed as an operon (the *aapA-F* operon), as there is little intergenic sequence between them (Fig. 1). The transmembrane domain (TMD) polypeptides (encoded by *PMI36_04621* and *_04620; aapBC*) are predicted to have six transmembrane domains each (http://www.cbs.dtu.dk/services/TMHMM-2.0/), placing this transporter in the type 1 family of ABC importers (29, 30). Because a similarly annotated ABC-type peptide transporter is adjacent to the *pipR* homolog in *E. meliloti* (18) (as well as several bacterial isolates from *Populus* roots [2, 21, 31]) and because PipR responds to the tripeptide SHS, we wondered whether the putative transporter was required for the PipR signal(s) to enter the cell. To assess the role of *aapB-F* in *pipA* activation, we created an in-frame deletion mutation in *aapB.* This AapB mutant did not respond to leaf macerates, peptone, or the SHS tripeptide (Fig. 4). The *aapB* mutation could be complemented with an *aapB* expression plasmid (see Fig. S2b in the supplemental material). These data are consistent with the idea that the PipR signal is taken up by cells via the *aap* operon-encoded ABC-type transporter.

### Peptidase mutants exhibit an enhanced *pipA-gfp* response.

**FIG 5:**
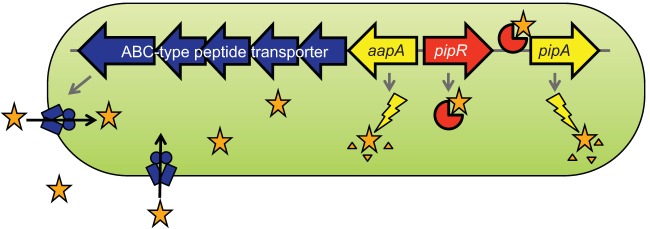
A model for PipR activation of *pipA* in GM79. The unknown signal(s) from plant macerates or peptone (stars) are taken up via the ABC-type transporter (4-component blue complex; the periplasmic-binding protein is not pictured). Once inside the cell, the signal can bind PipR, converting it to a form capable of binding the *pipA* promoter region and activating *pipA* and, possibly, *aapA*, resulting in high expression levels of peptidases (yellow lightning bolts). We hypothesize that these two peptidases act on the signal(s) or a bacterium-derived version of the signal(s) to reduce activity, thus creating a negative-feedback control loop.

We showed as described above that *aapA* and *pipA* encode peptidases capable of cleaving several different N-terminal amino acid residues (Table 1). We investigated whether peptidase gene inactivation had an effect on PipR signaling and found that pP*_pipA_-gfp* expression was much higher in the peptidase mutants than in the wild-type GM79 when grown with leaf macerate or peptone. When grown with leaf macerates, pP*_pipA_-gfp* expression in the peptidase single mutants and the *pipA aapA* double mutant was about twofold and sixfold higher, respectively, than in the wild type (Fig. 4a). These levels were even higher when cells were grown with peptone (2- to 5-fold higher for the single peptidase mutants and 14-fold higher in the *pipA aapA* double mutant relative to the levels in the wild type) (Fig. 4b). The higher pP*_pipA_-gfp* activities in the single *aapA* and *pipA* peptidase mutants were complemented to nearly wild-type levels by the expression of the respective peptidase gene (see Fig. S2c and d in the supplemental material). The AapA and PipA enzymes of GM79 are both predicted to localize to the cytoplasm (32). Our results are consistent with a model where the transported plant or peptone signals are degraded by the enzymatic activities of AapA and/or PipA (Fig. 5). However, we cannot exclude the possibility that the imported signal is modified by GM79 and that this modified form of the signal is a substrate for the peptidases or that the peptidases target other components of the PipR system.

## DISCUSSION

We show here that, as in several plant-associated bacteria (14–16, 18, 33), the *Populus* tree endophyte *Pseudomonas* sp. GM79 possesses a LuxR homolog that does not respond to AHL signals but instead recognizes an unknown compound in *Populus* leaf macerates. We call this LuxR homolog PipR. Our work demonstrates that PipR binds to a specific DNA sequence to activate the expression of its downstream proline iminopeptidase gene (*pipA*) in response to an unknown plant signal (Fig. 2b and 3). These results are similar to those found previously in *X. oryzae* (16, 24).

To extend our work in *Pseudomonas* sp. GM79 beyond what is known about the homologous *Xanthomonas* systems (14–17), we examined whether the genes surrounding *pipR* contribute to its activity. These flanking genes are annotated as being involved in peptide degradation and transport, leading us to hypothesize that PipR could respond to peptidelike compounds. Indeed, we found that a variety of peptide-rich peptones (including Bacto-peptone) and a specific tripeptide (SHS) could activate a PipR-dependent reporter.

A strain with a mutation in a transmembrane domain (TMD) protein gene (*aapB*) of the ABC transporter near *pipR* (Fig. 1) did not respond to plant leaf macerates, peptone, or the SHS tripeptide (Fig. 4), suggesting that these signal(s) enter cells by active transport. Transporters are not required for entry of AHL signals into cells, as AHLs can diffuse into and out of bacterial cells (34, 35). However, ABC-type transporters are used in many of the Gram-positive quorum-sensing systems for the import of peptide pheromone signals (reviewed in Cook and Federle [36]). There are no ABC-type transporters genetically linked to the *oryR* homologs in *Xanthomonas* species (http://img.jgi.doe.gov); however, upstream from the *oryR*-type genes is a gene annotated as a member of the amino acid/polyamine/organocation (APC) transporter superfamily (TC 2.A.3); interestingly this transporter gene is highly expressed (12-fold higher than in the wild type) in an *X. axonopodis* strain overexpressing an OryR (XagR) homolog (14). One could imagine that this APC transporter may play a role in *Xanthomonas* species similar to that of the GM79 ABC transporter: import of the OryR-responsive plant signal(s).

Strains with mutations in the flanking peptidase genes showed elevated expression of pP*_pipA_-gfp* compared to the level in the wild type when grown in the presence of leaf macerates and peptone (Fig. 4a and b). A similar result, increased *pip* expression compared to the level in the wild type, was reported for an *X. campestris* Pip^−^ mutant (15).

One interpretation of these results is that the peptidases enzymatically degrade the PipR signal(s) and in the peptidase mutants, less signal degradation occurs, resulting in higher PipR-dependent gene activation. A model of the PipR system consistent with these data is depicted in Fig. 5. Signal(s) enter the cell via the ABC-type transporter and activate PipR-dependent transcription of *pipA.* Although the Pip activity from *X. campestris* has been reported as localized to the periplasm (15), both AapA and PipA of *Pseudomonas* sp. GM79 are predicted to be cytoplasmic (32). For *Pseudomonas* sp. GM79, our data suggest that AapA and PipA can utilize a transported PipR ligand as a substrate, although we cannot exclude the possibility that they act on a compound derived from the ligand or on some other component of the PipR signaling system. This arrangement constitutes a negative-feedback loop for the system, which would ensure a rapid inactivation of *pipA* transcription when the signal becomes limited.

There is increasing evidence that not all orphan LuxR homologs sense AHLs. In addition to the plant-responsive OryR-type transcription factors discussed here, the LuxR homologs CarR (*Serratia* sp. strain 39006) (37) and MalR (*Burkholderia thailendensis*) (38), which both retain all of the conserved amino acid residues in the AHL-binding domain of LuxR homologs, do not require an AHL for activity. There are also examples of orphan LuxR homologs that utilize endogenous non-AHL compounds as signal ligands, including PluR (*Photorhabdus luminescens*) (9) and PauR (*Photorhabdus asymbiotica*) (10), which respond to α-pyrones and dialkylresorcinols, respectively. In addition, activators of AHL-responsive LuxR homologs have been identified which bear little resemblance to the native AHL signal ligand (39). Our work suggests that the GM79 PipR ligand is peptidelike. It will be interesting to purify and elucidate the structures of the PipR signals from both the plant macerate and peptone material. We predict that the plant and peptone signals will be structurally similar but not necessarily identical.

We are curious to test whether the PipR system mutants created here are also impaired in *Populus* host interactions, as is the case with PipR homologs in several plant pathogens (14–17) and mutualists (12, 18). We are also interested to know which GM79 genes, other than the peptidase genes, are under the control of PipR. In other bacteria, PipR homologs regulate not only proline iminopeptidase gene expression but additional traits, including those important for colonization of and movement through the plant host (motility [40] and biosurfactant and adhesin production [14]), accumulation of osmoprotectants (14), and synthesis of antifungal compounds (12).

PipR homologs are encoded in the genomes of several plant-associated bacterial genera, including *Xanthomonas*, *Dickeya*, *Agrobacterium*, *Rhizobium*, *Ensifer*, and *Pseudomonas* (reviewed in references 5, 11, and 12), and whether or not all these transcription factors respond to the same plant signal or different but related compounds is not known. The plant-responsive OryRs are of general importance, as they appear to play a role in the health of economically important plants (14–17). We believe *Pseudomonas* GM79 is a useful model to begin to understand the chemistry of what may prove to be a new family of interkingdom signals, or cues, involved in plant-bacterium interactions.

## MATERIALS AND METHODS

### Bacterial strains and growth conditions.

The bacterial strains and plasmids used are described in Table S1 in the supplemental material. *Pseudomonas* sp. GM79 and its derived strains were grown in R2A or M9 minimal medium (41) with 10 mM succinate (M9-suc) at 30°C. *E. coli* strains were grown in LB broth (42) and incubated at 37°C with shaking. Antibiotics were used when required at the following concentrations: 50 µg/ml (*Escherichia coli*) or 25 µg/ml (GM79) kanamycin, 100 µg/ml ampicillin, 20 µg/ml (*E. coli*) or 50 µg/ml (GM79) gentamicin, and 10 µg/ml tetracycline.

### Chemicals.

AHL signals were tested at 1 µM concentrations and included *N*-butanoyl-l-homoserine lactone (C4-HSL); *N*-3-oxo-hexanoyl-l-HSL (3-oxo-C6-HSL), *N*-3-oxo-octanoyl-l-HSL (3-oxo-C8-HSL), *N*-3-hydroxyoctanoyl-l-HSL (3-hydroxy-C8-HSL), *N*-3-oxododecanoyl-l-HSL (3-oxo-C12-HSL), and *N*-(*p*-coumaroyl)-l-HSL (*p*-coumaroyl-HSL) (purchased from Sigma-Aldrich, St. Louis, MO, or the University of Nottingham, Nottingham, United Kingdom). The β-naphthylamide and *p*-nitroanilide amino acid substrates were purchased from Sigma-Aldrich. Bacto-peptone, Bacto-soytone, and Bacto-tryptone were purchased from Becton, Dickinson, and Company (Franklin Lakes, NJ). The HS dipeptide was purchased from both AnaSpec (Fremont, CA) and Sigma-Aldrich. The tripeptides HSS, SHS, and SSH were custom synthesized by Peptide 2.0 (Chantilly, VA).

### Reporters, mutants, and plasmids.

All plasmids and primer sequences are described in Tables S1 and S2, respectively, in the supplemental material. We created the reporter plasmids pP*_pipA_-gfp* and pP*_aapA_-gfp* by PCR amplifying 263-bp DNA fragments containing the intergenic promoter regions, using GM79 genomic DNA as the template, and cloning the PCR products into HindIII-BamHI-digested pPROBE-NT (43). To create pP*_pipAmut_-gfp*, we ordered a gBlock gene fragment (Integrated DNA Technologies, Coralville, IA) containing the exact promoter sequence that was cloned into pP*_pipA_-gfp*, except that the CT nucleotides present in the predicted PipR-binding site were changed to TA. Mutant constructions were performed similarly: DNA sequences of about 500 bp from both up- and downstream of the desired in-frame deletion locations were either created by two-step overlap extension PCR amplification (Δ*pipA* mutation) or synthesized as a single DNA fragment of about 1 kb (Eurofins Genomics, Huntsville, AL) and cloned into EcoRI-BamHI-digested suicide vector pEX19-Gm (44). The knockout suicide vector was introduced into *Pseudomonas* GM79 strains by conjugal mating, and single-crossover mutants were selected by plating on M9-suc agar containing gentamicin. Double-crossover mutants were selected by streaking onto R2A agar containing 5% sucrose and screened for loss of Gm^r^.

For complementation of the *pipR* mutant, we PCR amplified a DNA fragment containing 250 bp of the *pipR* promoter sequence, the *pipR* gene, and the intergenic region between *pipR* and *pipA* and cloned the PCR product into HindIII-BamHI-digested pPROBE-NT (43). For complementation of the *pipA* mutant, the *pipA* gene and 254 bp of its promoter sequence were PCR amplified by using GM79 genomic DNA as the template, and the product was cloned into the BamHI-HindIII sites of pMMB67EH-TetRA. The plasmid for *aapA* complementation was constructed similarly except that only 190 bp of its promoter sequence was included. Because the *aapB* gene likely shares a promoter with the upstream *aapA* gene, we used the same forward primer as was used for complementation of the *aapA* mutant (AapCompFOR) plus a reverse primer for the 3′ end of the TMD gene (TsptCompREV) and used genomic DNA from the *aapA* mutant (79ΔAapA strain; see Table S1 in the supplemental material) as a PCR template. The PCR product was cloned into BamHI-HindIII-digested pMMB67EH-TetRA. Complementing plasmids (or pMMB67EH-TetRA vector controls) were introduced into the appropriate mutant strains harboring the pP*_pipA_-gfp* reporter by conjugal mating. All mutant and plasmid constructs were confirmed by DNA sequencing.

### Purification of His_6_-tagged proteins.

To obtain purified PipA and AapA, the genes were cloned into the His_6_-tagged protein expression vector pQE-30, creating plasmids pQEpipA and pQEaapA, respectively (see Tables S1 and S2 in the supplemental material). *E. coli* M15 pRep4 containing either pQEpipA or pQEaapA was grown at 30°C in 500 ml of LB plus antibiotics to an optical density at 600 nm of 0.6 (OD_600_). The production of His-tagged protein was then induced by the addition of 1 mM isopropyl-β-d-thiogalactopyranoside (IPTG) and incubation was continued at 16°C overnight, after which cells were pelleted, resuspended in buffer (50 mM NaH_2_PO_4_, 300 mM NaCl, 10 mM imidazole, pH 8), broken by French pressure cell, and centrifuged for 20 min at 14,000 × *g*. The His_6_-tagged proteins were purified from clarified cell extracts by cobalt resin column chromatography (Qiagen, Valencia, CA).

### Peptidase assays.

Enzyme assays were performed in 0.1-ml volumes containing 50 mM Tris, pH 7.4, 10 mM MnCl_2_, 0.75 mM amino acid substrate, and 0.6 µg His-tagged protein. Reaction mixtures were incubated for 20 min at 30°C and stopped by equivolume addition of 0.1 M acetic acid. Substrate cleavage was assessed by measuring either fluorescence (excitation at 355 nm and emission at 415 nm) for the β-naphthylamine-linked substrates or color [410 nm, molar extinction coefficient(M^−1^ cm^−1^) = 8,000] for the *p*-nitroanilide-linked substrates.

### Reporter assays.

Bioassays were performed in M9-suc for two reasons. (i) OryR accumulated in *X. oryzae* when grown in rich medium (peptone-yeast extract-salts) in the absence of plant macerates (24), suggesting that something in complex medium can induce the system. Therefore, we decided to use a minimal medium so as not to confound our results. (ii) Succinate was chosen as the carbon and energy source in the minimal medium because there were no significant growth rate differences between the wild-type and *pipR* mutant strains in this medium. Strains containing pP*_pipA_-gfp* were incubated overnight (24 h) in M9-suc plus kanamycin at 30°C with shaking. Cells were diluted 1:100 into fresh medium, 150-µl aliquots were added to individual wells of a 96-well microtiter dish containing 7.5 µl (except as indicated in Fig. 3) of material to be tested (leaf macerates, peptone, peptides, or AHLs), and the plates were sealed with Breathe-Easy sealing membrane (Research Products International, Mount Prospect, IL) and incubated at room temperature for ~24 h. GFP fluorescence (excitation at 485 nm and emission at 535 nm) and growth (OD_595_) were assessed using a Tecan Genios pro plate reader, and data were plotted as relative fluorescence units (RFU) per OD unit.

### Preparation of partially purified *Populus* leaf macerates and peptone material.

Because various additions to the bioassay strain culture showed both inhibitory (leaf macerates) and stimulatory (Bacto-peptone) growth effects, we developed a two-step cleanup protocol to produce the partially purified material used in all of our experiments. For leaf macerates, 5 g of *P. deltoides* WV94 leaves (greenhouse grown) were frozen in liquid nitrogen, macerated with a mortar and pestle, added to 100 ml of Milli-Q water (5% weight/vol), sterilized by autoclaving, and then filtered to remove plant tissue (as described in reference 24). Peptone was prepared in Milli-Q water at a concentration of 10 g/100 ml (10% wt/vol). Both leaf and peptone material were then passed over a C_18_ reverse-phase (RP) solid-phase extraction (SPE) cartridge (Waters Corp., Milford, MA). The C_18_-RP cartridge did not bind the active material but did retain a large amount of nonactive material (including the bacterial-growth-inhibiting activity in the leaf macerates). The flowthrough fraction was passed through an Amicon ultra-15 filter with a nominal molecular weight limit of 3,000 (Merck Millipore, Cork, Ireland) to remove any higher-mass, nonactive compounds. Partially purified material was concentrated, resuspended in Milli-Q water to its original concentration, and filter sterilized with a 0.2-µm syringe filter.

### Peptide screening with Biolog plates.

Biolog phenotype microarray plates for nitrogen utilization assays (PM6, PM7, and PM8) were used (Biolog, Inc., Hayward, CA). GM79 (pP*_pipA_-gfp*) cells in M9-suc medium were incubated in the Biolog plates for 18 h, and then GFP fluorescence (excitation at 485 nm and emission at 535 nm) and growth (OD_595_) were determined. As a control for PipR activity, 1% peptone was added to the l-glutamine positive control present on every Biolog plate.

## SUPPLEMENTAL MATERIAL

Figure S1  Amino acid sequence alignment of *Pseudomonas* sp. GM79 LuxR homologs with other representative LuxR family members. Included in the alignment are known AHL-responsive (*Agrobacterium tumefaciens* TraR, *Vibrio fischeri* LuxR, and *Pseudomonas aeruginosa* LasR) and plant-responsive LuxR homologs (*Xanthomonas oryzae* OryR and *Ensifer meliloti* NesR). The asterisks indicate the amino acid residues that are conserved among all LuxR^−^ (including the plant-responsive LuxR) homologs. The two residue positions boxed in red are residues that vary between the plant-responsive LuxR homologs, OryR and NesR, and the AHL-responsive LuxRs (11, 12). Sequences shaded in black are identical between all seven sequences; those shaded in grey indicate amino acid similarity. PipR from GM79 only varies in one of these positions. Amino acid sequences used for the alignment are available from the Joint Genome Institute Integrated Microbial Genomes (IMG) website (http://img.jgi.doe.gov/cgi-bin/w/main.cgi) under the following identification (ID) numbers: PipR (PMI36_04623), IMG Gene ID 2511364843; PpoR (PMI36_01833), IMG Gene ID 2511362058; OryR, IMG Gene ID 637631608; NesR, IMG Gene ID 637183639; TraR, IMG Gene ID 639300894; LuxR, IMG Gene ID 637638274; and LasR, IMG Gene ID 637051823. Clustal-Ω was used to align the sequences, and Boxshade was used to determine the degree of residue shading. Download Figure S1, EPS file, 1.1 MB

Figure S2  Mutations in *pipR*, *aapB*, *pipA*, and *aapA* are complemented with plasmid-borne copies of the wild-type gene. Strains are wild type (WT) and the Δ*pipR* mutant (PipR^−^) (a), wild type (WT) and the Δ*aapB* transporter mutant (AapB^−^) (b), wild type (WT) and the Δ*pipA* peptidase mutant (PipA^−^) (c), and wild type (WT) and the Δ*aapA* peptidase mutant (AapA^−^) (d), carrying the indicated plasmids. In panels b, c, and d, the pMMB67EH-TetRA vector control (pMMvector) is included for comparison. Activity of the *pipA-gfp* reporter grown in the presence of water control (white bars), 0.25% leaf macerates (green bars), or 0.5% peptone (orange bars) is shown as relative fluorescence units (RFU) per OD unit. The data are the mean values from six replicates, and the error bars represent the standard deviations. Download Figure S2, EPS file, 0.5 MB

Table S1  Strains and plasmids used in this study. Table S1, DOCX file, 0.1 MB

Table S2  Primers used in this study. Table S2, DOCX file, 0.1 MB
