# Supplementary material for: A LuxR Homolog in a Cottonwood Tree Endophyte That Activates Gene Expression in Response to a Plant Signal or Specific Peptides
Source: mBio. 2016 Aug 2;7(4):e01101-16. doi: 10.1128/mBio.01101-16 (PMC4981722; doi:10.1128/mBio.01101-16)
Supplement: Table S1 — Strains and plasmids used in this study. [file mbo004162917st1.docx]

Supplemental Table 1. Strains and plasmids used in this study

| Strain or plasmid | Relevant genotype/phenotype | Ref. |
| --- | --- | --- |
| Strains |  |  |
| *Pseudomonas sp.* |  |  |
| GM79 | Wild-type isolated from *Populus deltoides* root endosphere | ^1^ |
| 79ΔpipR | *pipR* (*PMI36_04623*) in-frame deletion in GM79 | this work |
| 79ΔaapB | transporter TMD gene (*aapB/PMI36_04621*) in-frame deletion in GM79 | this work |
| 79ΔpipA | *pipA* (*PMI36_04624*) in-frame deletion in GM79 | this work |
| 79ΔaapA | *aapA* (*PMI36_04622*) in-frame deletion in GM79 | this work |
| 79ΔpipAaapA | *pipA* (*PMI36_04624*) *aapA* (*PMI36_04622*) double deletion in GM79 | this work |
| *E. coli* |  |  |
| M15  S17-1 | F-, Φ80ΔlacM15, thi, lac-, mtl-, recA+  *recA*, *thi*, *pro,* RP4-2-Tc::Mu-Km::Tn7 | Qiagen  ^2^ |
|  |  |  |
| Plasmids |  |  |
| pQE30 | N-terminal His-protein expression vector, Ap^R^ | Qiagen |
| pRep4 | *lacI*–containing vector, Km^R^ | Qiagen |
| pQEpip | N-terminal His_6_-PipA expression plasmid, Ap^R^ | this work |
| pQEaap | N-terminal His_6_-AapA expression plasmid, Ap^R^ | this work |
| pPROBE-NT | Broad host vector containing promoterless *gfp* reporter, Km^R^ | ^3^ |
| pP*_pipA_-gfp* | *pipA* promoter region cloned into *gfp*-reporter pPROBE-NT, Km^R^ | this work |
| pP*_pipAmut_-gfp* | pP*_pipA_-gfp* with an PipR-binding sequence mutation, Km^R^ | this work |
| pR_P*_pipA_-gfp* | *pipA* promoter region plus the upstream *pipR* gene and its native promoter cloned into *gfp*-reporter pPROBE-NT, Km^R^ | this work |
| pP*_aapA_-gfp* | *aapA* promoter region cloned into *gfp*-reporter pPROBE-NT, Km^R^ | this work |
| pEX19Gm | Suicide vector, *sacB*, Gm^R^ | ^4^ |
| pMMB67EH-  TetRA | IPTG-inducible, broad host expression plasmid derived from pMMB67EH^5^, Tc^R^ | gift from S.I. Miller lab, |
| pMMaapB | transporter TMD gene (*aapB/PMI36_04621*) cloned into pMMB67EH-TetRA, Tc^R^ | this work |
| pMMpipA | *pipA* gene (*PMI36_04624*) cloned into pMMB67EH-TetRA, Tc^R^ | this work |
| pMMaapA | *aapA* gene (*PMI36_04622*) cloned into pMMB67EH-TetRA, Tc^R^ | this work |
|  |  |  |
|  |  |  |

SUPPLEMENTAL REFERENCES

1 Brown, S. D. *et al.* Twenty-one *Pseudomonas* genomes and ninteen genomes from diverse bacteria isolated from the rhizophere and endosphere of *Populus deltoides*. *J Bacteriol* **194**, 5991-5993 (2012).

2 Simon, R., Priefer, U. & Puhler, A. A broad host range mobilitization system for in vivo genetic engineering: transposon mutagenesis in Gram-negative bacteria. *Nature Biotech* **1**, 784-791 (1983).

3 Miller, W. G., Leveau, J. H. & Lindow, S. E. Improved *gfp* and *inaZ* broad-host-range promoter-probe vectors. *Mol Plant Microbe Interact* **13**, 1243-1250 (2000).

4 Tung, T., Karkhoff-Schweizer, R. R., Kutchma, A. J. & Schweizer, H. P. A broad-host-range Flp-*FRT* recombination system for site-specific excision of chromosomally-located DNA sequences: application for isolation of unmarked *Pseudomonas aeruginosa* mutants. *Gene* **212**, 77-86 (1998).

5 Furste, J. P. *et al.* Molecular cloning of the plasmid RP4 primase region in a multi-host-range *Ptac* expression vector. *Gene* **48**, 119-131 (1986).
