# Supplementary material for: A LuxR Homolog in a Cottonwood Tree Endophyte That Activates Gene Expression in Response to a Plant Signal or Specific Peptides
Source: mBio. 2016 Aug 2;7(4):e01101-16. doi: 10.1128/mBio.01101-16 (PMC4981722; doi:10.1128/mBio.01101-16)
Supplement: Table S2 — Primers used in this study. [file mbo004162917st2.docx]

Supplemental Table 2. Primers used in this study

| Name | DNA sequence (5’-3’) | Description |
| --- | --- | --- |
| P*_pipA_-gfp*FOR | GGCAAGCTTGGGCGGCGGAGGGGCTGAC | *pipA-gfp* promoter reporter |
| P*_pipA_-gfp*REV | CGCGGATCCAGATTCCATTTCTTTGTGC | *pipA-gfp* promoter reporter |
| P*_aapA_-gfp*FOR | GGCAAGCTTCGTTGTCCATCTGCTCATCC | *aapA-gfp* promoter reporter |
| P*_aapA_-gfp*REV | CGCGGATCCACGCCACATCGCAACTCTCC | *aapA-gfp* promoter reporter |
| PipRcompFOR | GGCAAGCTTCACGCGCAACCCCTTGTCCT | 79ΔpipR complementation |
| TsptCompREV | GGCAAGCTTTCATCGGATTTTCACGCGTGG | 79ΔaapB complementation |
| PipAcompFOR | CGCGGATCCGGGCGGCGGAGGGGCTGAC | 79ΔpipA complementation |
| PipAcompREV | GGCAAGCTTTCATGCCCCAGGACAGG | 79ΔpipA complementation |
| AapAcompFOR | CGCGGATCCGCTCTGCATCCATTGCGCTCC | 79ΔaapA complementation |
| AapAcompREV | GGCAAGCTTCTAACCTCGGTGCCGCTGGAGG | 79ΔaapA complementation |
| HisPipAFOR | CCCGGATCCATGGAATCTATCGGAACTCG | His_6_-PipA expression |
| HisPipAREV | ACCAAGCTTTCATGCCCCAGGACAGG | His_6_-PipA expression |
| HisAapAFOR | CCCGGATCCATGTGGCGTGAAATCGAGC | His_6_-AapA expression |
| HisAapAREV | ACCAAGCTTCTAACCTCGGTGCCGCTGG | His_6_-AapA expression |
| PipAdelFor1 | GATGAATTCCGTTTCACCCTTTGTCTGGT | 79ΔpipA construction |
| PipAdelRev1 | AGGACAGGAAAGACGCGGGTCGAAGCCTTCGCGAGTTCCGAT | 79ΔpipA construction |
| PipAdelFor2 | GATGGATCCGGAGCCGAAAATACTGCTGA | 79ΔpipA construction |
| PipAdelRev2 | ATCGGAACTCGCGAAGGCTTCGACCCGCGTCTTTCCTGTCCT | 79ΔpipA construction |
